# Semi-dominant effects of a novel *ripening inhibitor* (*rin*) locus allele on tomato fruit ripening

**DOI:** 10.1371/journal.pone.0249575

**Published:** 2021-04-22

**Authors:** Yasuhiro Ito, Nobutaka Nakamura, Eiichi Kotake-Nara

**Affiliations:** Food Research Institute, National Agriculture and Food Research Organization (NARO), Tsukuba, Ibaraki, Japan; Hirosaki University Graduate School of Medicine, JAPAN

## Abstract

The tomato (*Solanum lycopersicum*) *ripening inhibitor* (*rin*) mutation completely represses fruit ripening, as *rin* fruits fail to express ripening-associated genes and remain green and firm. Moreover, heterozygous *rin* fruits (*rin*/+) ripen normally but have extended shelf life, an important consideration for this perishable fruit crop; therefore, heterozygous *rin* has been widely used to breed varieties that produce red tomatoes with improved shelf life. We previously used CRISPR/Cas9 to produce novel alleles at the *rin* locus. The wild-type allele *RIN* encodes a MADS-box transcription factor and the novel allele, named as *rinG2*, generates an early stop codon, resulting in C-terminal truncation of the transcription factor. Like *rin* fruits, *rinG2* fruits exhibit extended shelf life, but unlike *rin* fruits, which remain yellow-green even after long-term storage, *rinG2* fruits turn orange due to ripening-associated carotenoid production. Here, to explore the potential of the *rinG2* mutation for breeding, we characterized the effects of *rinG2* in the heterozygous state (*rinG2/+*) compared to the effects of *rin*/+. The softening of *rinG2/+* fruits was delayed compared to the wild type but to a lesser degree than *rin/+* fruits. Lycopene and β-carotene levels in *rinG2/+* fruits were similar to those of the wild type, whereas *rin/+* fruits accumulated half the amount of β-carotene compared to the wild type. The *rinG2/+* fruits produced lower levels of ethylene than wild-type and *rin/+* fruits. Expression analysis revealed that in *rinG2/+* fruits, the *rinG2* mutation (like *rin*) partially inhibited the expression of ripening-associated genes. The small differences in the inhibitory effects of *rinG2* vs. *rin* coincided with small differences in phenotypes, such as ethylene production, softening, and carotenoid accumulation. Therefore, *rinG2* represents a promising genetic resource for developing tomato cultivars with extended shelf life.

## Introduction

Extending the shelf life of agricultural products is important for supplying fresh, nutritious foods and reducing food losses. Storing fruits and vegetables at low temperature and maintaining an appropriate atmosphere during transport and storage can preserve the quality of fresh vegetables and fruits. Developing cultivars that produce fruits with resistance to over-ripening is another effective way to maintain fruit quality [1]. Tomato (*Solanum lycopersicum*), a major vegetable consumed all over the world, is a typical fruit species that is often wasted due to over-ripening-associated quality losses, such as cracking, wilting, and infection [2, 3]. Therefore, extending shelf life has been an important objective for tomato breeding programs [4–6].

**Fig 1:**
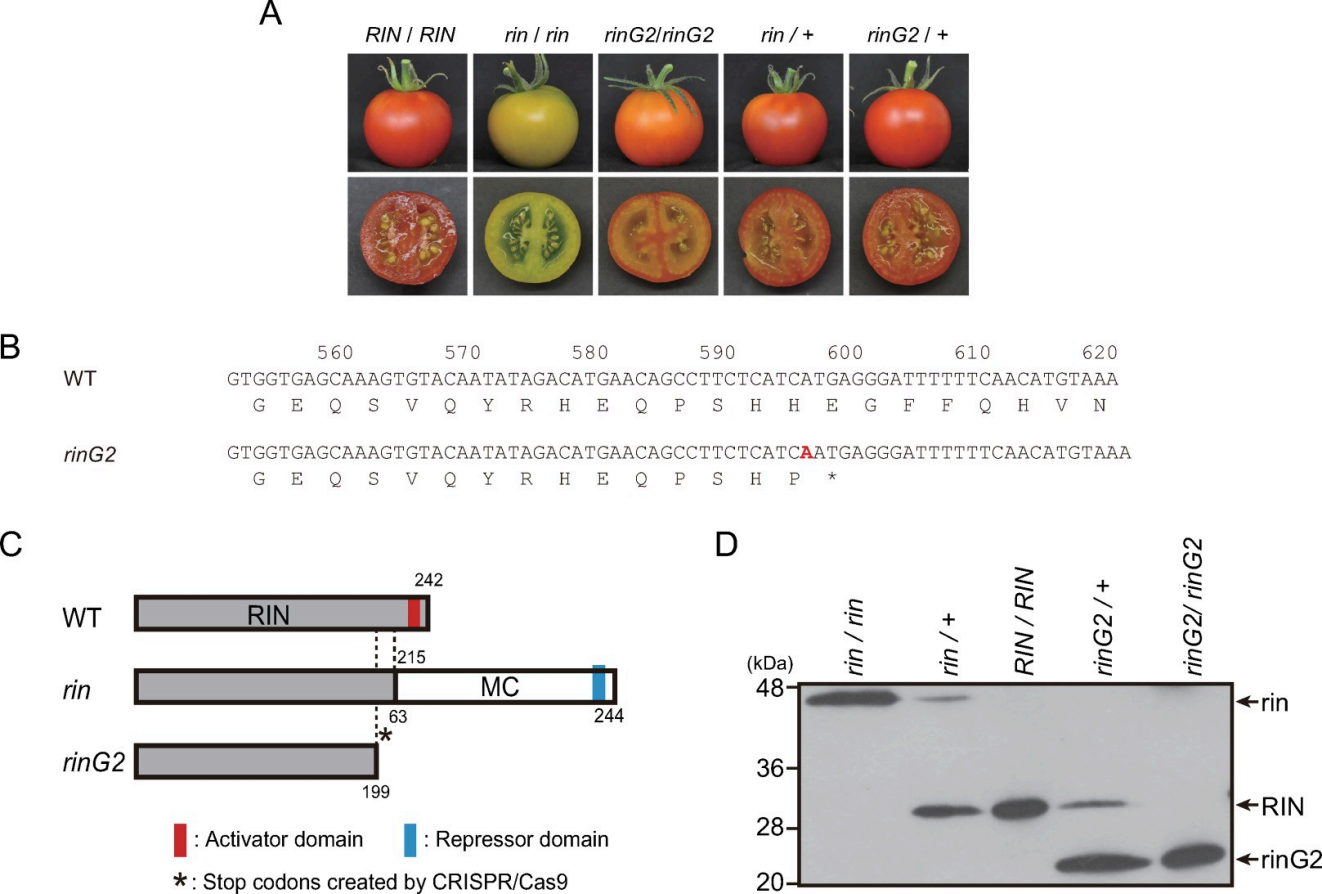
Expression of the *rinG2* allele in tomato. **A.** Ripe fruits with homozygous or heterozygous mutant alleles in the *rin* locus. Fruits with the indicated genotypes were harvested at 7 days after the breaker stage. **B.** The *rinG2* mutation generates an early stop codon. “A” in red represents the base inserted by genome editing. **C.** Schematic diagrams of the proteins encoded by the *rin* and *rinG2* alleles. The *rin* allele produces a protein encoded by a fusion mRNA composed of *RIN* lacking the last exon and *MC* lacking the first exon [9]. The *rinG2* mutation generates an early stop codon, which removes the C-terminus including the transcriptional activator domain of RIN [23]. **D.** Accumulation of mutant proteins in heterozygous fruits. In *rin-* and *rinG2-*heterozygous fruits, the mutant proteins (indicated as rin and rinG2, respectively) accumulated simultaneously with wild-type protein (RIN). Proteins from nuclei of fruits harvested at 4 days after the breaker stage were separated and subjected to an immunoblotting assay with RIN-antibodies [15]. We did not measure the detection efficiencies of the allelic proteins in extracts from heterozygous lines, and therefore, we cannot conclusively state whether the differences in signal intensities between the allelic proteins reflect differences in their abundances in the cells.

Various mutations affecting fruit physiology in tomato have been discovered, and a variety of ripening mutants have been investigated as genetic resources to develop cultivars that produce fruits with extended shelf life [7, 8]. The major ripening mutants *ripening inhibitor* (*rin*), *non-ripening* (*nor*), and *Colorless non-ripening* (*Cnr*), which exhibit completely inhibited ripening, have been extensively studied. These loci encode transcription factors of the MADS-box family, NAC family, and SBP-box family, respectively [9–11]. The *rin* and *nor* mutants exhibit semi-dominant phenotypes in terms of ripening physiology: the fruits of the F_1_ hybrids between each mutant and wild type turn red at the ripening stage but show delayed softening (Fig 1A) [6, 12, 13]. The *alcobaca* (*alc*) mutant, an allele of *nor*, also exhibits a semi-dominant phenotype [5].

The *rin* mutant has been extensively used for studying the regulation of the ripening process. The *RIN* locus encodes a SEPALLATA type MADS-box transcription factor with transcription-activating activity [9, 14]. *RIN* is specifically expressed at the onset of ripening and during ripening; RIN forms MADS-box transcription factor complexes that bind to the promoter regions of thousands of ripening-associated genes [15–21]. Recent studies using CRISPR/Cas9-mediated mutagenesis of *RIN* have clarified the precise role of RIN as a ripening regulator [22–25]. In contrast to the non-ripening phenotype of the *rin* mutant, *RIN* knockout mutant fruits undergo initial ripening, accumulating small amounts of lycopene and increasing ethylene production at a low but detectable level, suggesting that RIN is required for full ripening but not for the initiation of ripening [22, 24]. Surprisingly, RIN-knockout mutant fruits softened excessively, even compared to wild-type fruits, suggest that RIN does not simply accelerate ripening-associated changes but also represses excess softening [23].

Another allelic mutant in the *RIN* locus, *rinG2*, was also developed by CRISPR/Cas9 and exhibited another specific ripening phenotype. Similar to *rin*, the *rinG2* mutation confers delayed softening compared to the wild type, but unlike *rin* fruits, *rinG2* fruits show ripening-specific changes in coloration, turning orange due to the accumulation of carotenoids (Fig 1A) [23]. The phenotype of *rinG2* offers great promise for use in breeding tomato cultivars with extended shelf life; however, the low lycopene accumulation in these fruits must be improved.

In the current study, to examine the possible use of the *rinG2* mutation as a practical method for breeding tomatoes with extended shelf life, we investigated the heterozygous effects of *rinG2* by developing F_1_ hybrid plants with the *rinG2/+* genotype. Similar to the classic *rin* mutation, *rinG2* had semi-dominant effects on ripening physiology. We compared the phenotypes of *rinG2/+* vs. *rin/+* and explored the possible molecular activities of allelic mutant proteins in the cells of heterozygous fruits.

## Materials and methods

### Plants with mutations in the *rin* locus

The tomato (*Solanum lycopersicum*) cultivar Ailsa Craig (AC), which was used as the parental line to produce the genome-edited mutants, was used as the wild-type control. The *rinG2* mutation was generated previously with the CRISPR/Cas9 system [26]. The T_1_ generation line G2#13–15, a *rinG2* homozygous mutant, was crossed with AC to produce an F_1_ hybrid plant with the *rinG2/RIN* genotype (*rinG2/+*). LA3754 is a *rin* mutant line with a near-isogenic AC background. Plants were grown in a growth room under a 16-h-light (fluorescent lamps)/8-h-dark cycle at 25°C.

### Immunoblotting analysis

Nuclear extracts from ripening fruits were prepared for SDS-PAGE as previously described [14]. Nuclear extracts were separated by SDS-PAGE and electrophoretically transferred to Immobilon-P PVDF membrane (Merck). The preparation of polyclonal anti-RIN antibodies was described previously [15]. Immunodetection reactions were performed on membranes with antiserum against RIN as primary antibodies and horseradish peroxidase (HRP)-linked anti-rabbit IgG (GE Healthcare) as secondary antibody. Fluorescence signals generated with the Chemi-Lumi One L (Nacalai Tesque) were detected by exposure to X-ray film.

### Characterization of fruit phenotypes

The fruits were stored at 25°C and their firmness was measured with a Tensipresser IIX texture analyzer (Taketomo Electric). Firmness was determined based on the force required to reduce fruit diameter by 5% with a 10-mm diameter plunger.

Carotenoid contents were measured in fruits harvested at 10 days after the day of ripening initiation. Carotenoid extraction was performed as described previously [27]. Briefly, 0.5 g of flesh from the equatorial region of a fruit was sampled and stored at –80°C. The frozen tissue was homogenized in 2.5 mL chloroform and 1 mL methanol using an Ultra-Turrax T25 homogenizer (IKA). The homogenate was mixed with 1 mL Tris-HCl (pH 8.0) and stored on ice for 20 min. After centrifugation at 3,000 ×*g* for 5 min in a himac centrifuge CR21G (Hitachi), the organic lower phase containing carotenoids was retrieved. The residual carotenoid was re-extracted from the aqueous phase with 2.5 mL chloroform. After centrifugation at 3,000 ×g for 5 min, the lower organic phase was retrieved and mixed with the previously generated carotenoid preparation. Carotenoids were quantified by HPLC using an LC-20AT pump, an SPD-M10A photodiode array detector, and a CTO-10AS column (Shimadzu) on an ODS-80Ts column (2.0 × 250 mm, Tosoh) with an ODS-S1 precolumn (2.0 × 10 mm, Tosoh). Isocratic analysis was performed at a flow rate of 0.2 mL/min with methanol/ethyl acetate (70:30, v/v) containing 0.1% ammonium acetate. Lycopene was quantified from the peak area at 470 nm, and β-carotene was quantified from the peak area at 450 nm.

To measure ethylene production, a fruit was enclosed in a 1-L chamber for 2 h (for wild type and the heterozygous mutant lines) or 5 h (for the *rin* and *rinG2* mutant lines). A 1-mL sample of headspace gas was withdrawn from the chamber. The gas was injected into a GC8A gas chromatograph (Shimadzu), and the ethylene peak was determined with a C-R8A Chromatopac (Shimadzu).

### Reverse-transcription quantitative PCR (qRT-PCR)

Flesh in the equatorial region of a fruit was sampled and stored at –80°C. The tissue was ground in liquid nitrogen, and total RNA was extracted from the ground tissue using an RNeasy Plus Kit (Qiagen). The RNA was reverse-transcribed into cDNA using ReverTra Ace qPCR RT Master Mix (Toyobo). Quantitative PCR amplification was conducted using Thunderbird SYBR qPCR Mix (Toyobo) with a CFX Connect Real-Time PCR Detection System (Bio-Rad). Relative quantification of the transcript level of each gene was performed using the 2^-ΔΔCT^ method [28]. The clathrin adaptor complex medium subunit gene (*CAC*) was used as a reference [29]. Primer sequences are listed in S1 Table.

## Results

### The *rinG2* mutation produces an early stop codon resulting in the accumulation of truncated RIN protein in heterozygous fruit

The classic *rin* mutant allele encodes a fusion of RIN with Macrocalyx (MC), which is encoded at the genomic region adjacent to *RIN* [9, 30] (Fig 1C). The *rinG2* allele was developed by genome editing of the *RIN* gene [23, 26]. Genome editing resulted in a one-base insertion at position 596 from the translation start position in the mRNA, which caused a frame-shift producing an early stop codon just after the insertion (Fig 1B). This mutation modified the wild-type RIN (242 amino acid residues) into a peptide with 199 amino acid residues composed of 198 amino acids from the original RIN protein and an additional amino acid produced by the frame shift (Fig 1C). The truncation removed the transcriptional-activating domain from the wild-type RIN transcription factor, but the truncated rinG2 protein retained the ability to bind to RIN target sites by forming a MADS-box transcription factor complex [23].

Fruits of heterozygous plants produced by a cross between the *rinG2* mutant and wild type (*rinG2/+*) turned red, like those of wild type and heterozygous plants produced by crossing the *rin* mutant and wild type (*rin/+*, Fig 1A). Immunoblotting analysis with RIN-specific antiserum detected the wild-type RIN protein as well as the RIN–MC fusion protein encoded by the *rin* mutant allele and the truncated protein encoded by the *rinG2* allele (Fig 1D). In fruits of heterozygous plants with the genotypes *rin/+* and *rinG2/+*, the mutant proteins encoded by the *rin* and *rinG2* alleles were detected simultaneously with wild-type protein, suggesting that the mutant proteins play specific roles in the cells of heterozygous fruit. Previous studies demonstrated that rin protein has transcriptional repressor activity, whereas wild-type RIN protein has transcriptional activator activity, and rinG2 protein has neither activator nor repressor activity, although the rinG2 protein still binds to target genome regions and forms a MADS-box transcription factor complex, as does wild-type RIN protein [22, 23].

### Shelf life of *rinG2*/+ fruits

We evaluated the phenotypes of the fruits during long-term storage. To normalize the fruit stages among lines, we defined the day when a fruit showed the first signs of red pigmentation as the initiation of fruit ripening, which is termed the breaker stage. The transition into the breaker stage (red pigmentation) was clearly recognized in the fruits of the *rin*/+ and *rinG2/+* plants, as well as wild-type fruits. The *rinG2/rinG2* fruits also showed some red pigmentation, although the coloring was relatively faint. The *rin/rin* fruits never accumulated red pigments but did transition into the mature green stage, i.e., the stage just before the breaker stage: wild-type fruits showed reduced green pigmentation and a faint yellowish color during this stage [18, 23]. For *rin/rin* fruits, the stage at 3 days after the mature green stage was defined as the breaker-equivalent stage.

**Fig 2:**
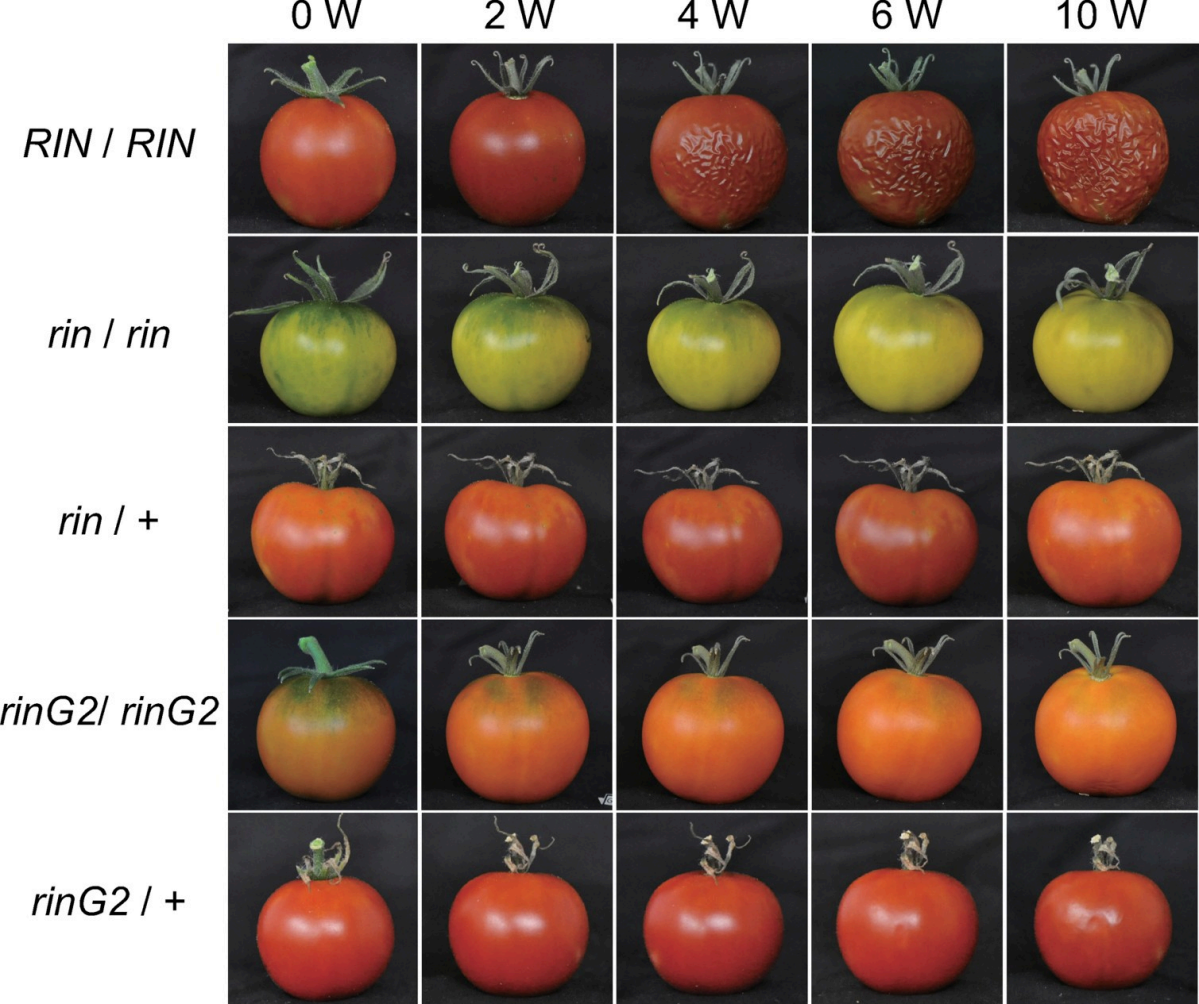
Heterozygous effects of *rinG2* on fruit shelf life. Fruits were harvested at 7 days after the breaker stage (0 W) and stored at 25°C.

We harvested fruits from all lines at 7 days after the breaker stage and stored them at 25°C (Fig 2). During storage conditions, the surfaces of wild-type fruits became wrinkled beginning at 2 weeks after the breaker stage. These fruits gradually dried up and became smaller. By contrast, the *rin/rin* and *rinG2/rinG2* fruits maintained their appearance over a 10-week period, as did the *rin*/+ fruits. The surfaces of *rinG2/+* fruits began to wrinkle after 6 weeks, but this process was delayed and was less severe than that observed in the wild-type fruits (Figs 2 and S1).

**Fig 3:**
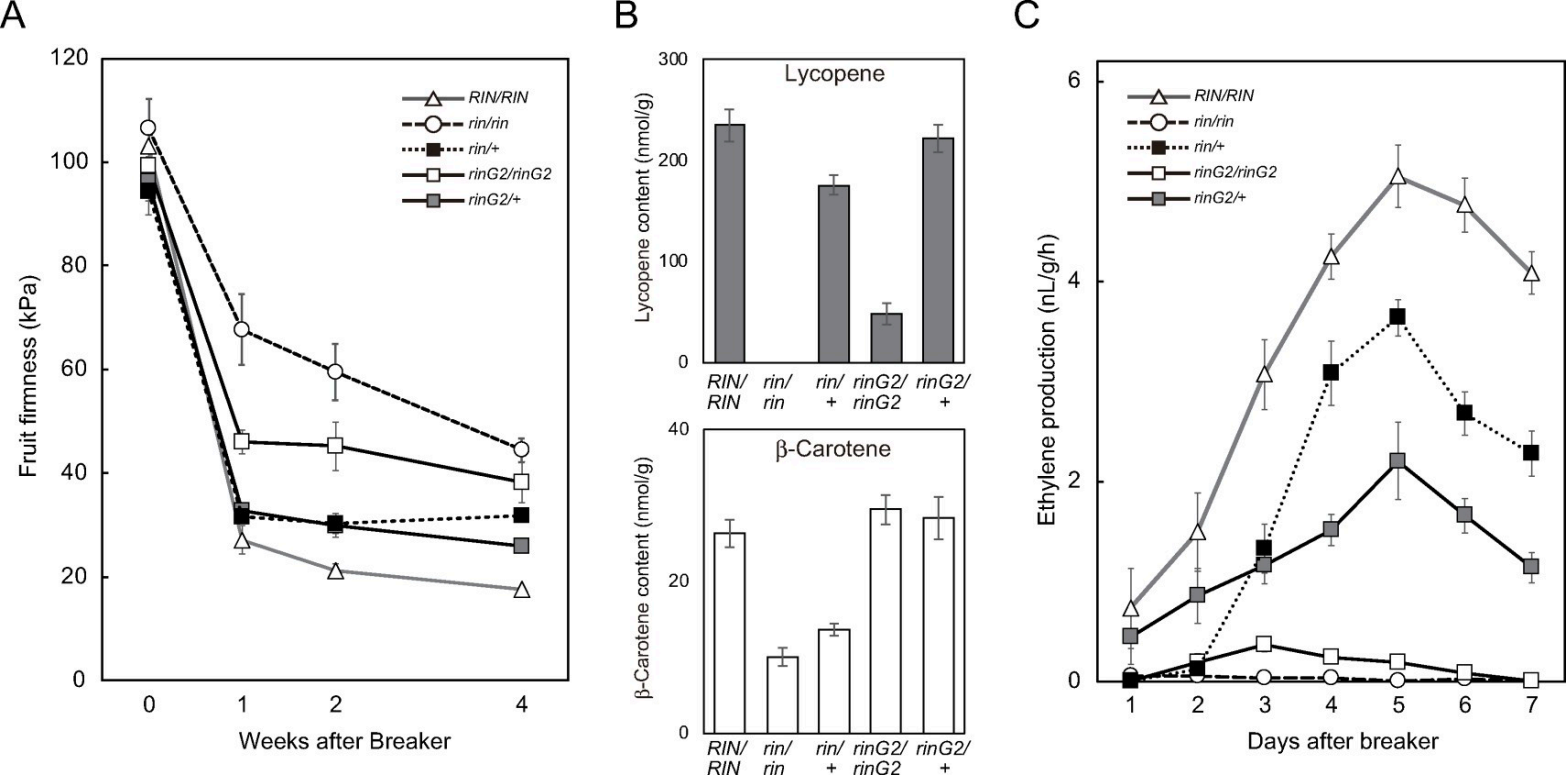
Fruit softening, carotenoid accumulation, and ethylene biosynthesis in *rinG2/+*. **A.** Firmness of fruits with mutations in the *rin* locus. Fruits harvested just before the breaker stage were examined as 0 week samples. Breaker stage fruits were harvested and examined at 1, 2, and 4 weeks after harvest. Data represent the means ± SE of five biological replicates. **B.** Lycopene and β-carotene contents of fruits with mutations in the *rin* locus. Fruits were harvested at 10 days after the breaker stage. Data represent the means ± SE of five or six biological replicates. **C.** Ethylene biosynthesis in fruits with mutations in the *rin* locus. Fruits just before the breaker stage were harvested and examined daily. Data represent the means ± SE of at least four biological replicates.

We measured fruit firmness by pressing the surfaces of fruits that had been harvested at the breaker stage and stored at 25°C (Fig 3A). Wild-type fruits showed the greatest reduction in firmness after 4 weeks of storage. The fruit firmness decreased rapidly during the first week and continued to gradually decrease thereafter. Under these conditions, *rin/rin* fruits were the hardest among the lines examined, followed by *rinG2/rinG2* fruits. The *rin/+* and *rinG2/+* fruits showed similar reductions in fruit firmness to the wild type after the first week of storage, but further reductions were delayed. The fruit-softening rate of *rinG2/+* fruits was similar to that of *rin/+* fruits for the first two weeks of storage; subsequently, *rinG2/+* fruits became slightly softer than *rin/+* fruits but were still harder than wild-type fruits.

**Fig 4:**
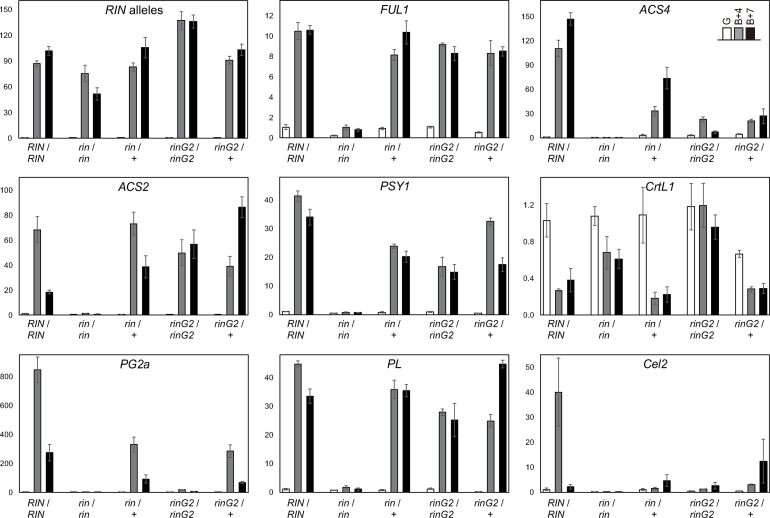
The expression of ripening-associated genes in *rinG2/+* fruit. mRNA levels were measured by qRT-PCR. mRNA was prepared from fruits at the green-coloring stage just before the initiation of ripening (G) and fruits during ripening (4 and 7days after the breaker stage; B+4 and B+7). A primer set for the *RIN* alleles anneals to a common sequence of the wild type, *rin*, and *rinG2* alleles. Data represent the means ± SE of three biological replicates.

To investigate ripening-specific gene expression, we prepared mRNA from fruits harvested at the pre-ripening stage (G) and at 4- and 7-days after the breaker stage (B+4 and B+7). Because the transcription of the *rin* mutant allele in the *rin* homozygous mutant is induced at a time equivalent to ripening initiation in wild-type fruits even though ripening-associated ethylene levels do not increase [23], the transcriptional induction of any alleles in the *rin* locus occurs as an initial reaction to ripening prior to the increase in ethylene levels. Therefore, the expression of these alleles can be regarded as a marker of the transition into the initial ripening stage. In each line, the expression of alleles in the *rin* locus was not detected at the G-stage but was intense during ripening (B+4 and B+7 stages), indicating that the fruits of each line, including the *rin* mutant, were harvested at equivalent stages (Fig 4).

*FRUITFULL1* (*FUL1*) encodes a MADS-box transcription factor that forms a complex with RIN and regulates ripening [17, 18, 31–33]. The ripening-associated upregulation of *FUL1* was not detected in the *rin* mutant, but it was detected in the *rinG2* mutant and the two heterozygous lines, pointing to the activity of the rin mutant protein as a transcriptional repressor (Fig 4). We then focused on three genes related to cell wall metabolism during ripening to look for relationships between their expression and aspects of fruit softening in the heterozygous lines (Fig 4). *Polygalacturonase2a* (*PG2a*) encodes an enzyme that hydrolyzes α-1,4 glycosidic bonds within pectins and is strongly induced during ripening [4, 34]. *PG2a* expression was correlated to the rate of fruit softening in all lines examined. Wild-type fruits showed intense *PG2a* expression during ripening, whereas its expression was strongly repressed in the *rin/rin* and *rinG2/rinG2* fruits. Both the *rin/+* and *rinG2/+* lines showed ripening-specific upregulation of *PG2a*, but the *PG2a* transcript level in these lines was approximately 39% and 34% of the wild type at the B+4 stage, and 34% and 25% of wild type at the B+7 stage, respectively.

Pectate lyase (PL), which depolymerizes pectin via a β-elimination mechanism, is a major contributor to tomato fruit softening [35, 36]. *PL* expression was observed specifically during ripening in wild-type fruits but was strongly repressed in *rin/rin* fruits. Unlike *PG2a*, *PL* was expressed at only a slightly lower level in *rinG2/rinG2* fruits and at a comparable level in both heterozygous lines vs. the wild type. *Cellulase 2* (*Cel2*), encoding an endo-β-1,4-glucanese, was specifically expressed during ripening in wild-type fruits. This gene was expressed at significantly lower levels in the homozygous and heterozygous *rin* and *rinG2* fruits compared to the wild type at the B+4 stage; among these, the *rinG2/+* fruits showed slightly higher expression at the B+7 stage compared to the other fruits.

### Carotenoid accumulation in *rinG2/+* fruits

We extracted carotenoids from fully ripened fruits that had been harvested at 10 days after the breaker stage and measured the accumulation of the pigments lycopene (red) and β-carotene (orange) (Fig 3B). The abundant accumulation of lycopene in wild-type fruits was completely inhibited by the *rin* mutation, whereas low but measurable levels of lycopene were detected in *rinG2/rinG2* fruits. The reduced accumulation of lycopene in the mutants was restored by crossing with wild-type plants, as *rin/+* fruits as well as *rinG2/+* fruits accumulated lycopene at levels comparable to those in wild-type fruits; lycopene levels were slightly higher in *rinG2/+* fruits vs. *rin/+* fruits. The β-carotene levels were also reduced by the *rin* mutation, but unlike lycopene, β-carotene levels in *rinG2/rinG2* fruits were similar to those of wild-type fruits. We also detected a difference in the two heterozygotes: *rin/+* fruits accumulated β-carotene at 52% of wild-type levels, whereas β-carotene levels in *rinG2/+* fruits were similar to those of the wild type.

Lycopene accumulation during ripening depends on the increased expression of enzymes that catalyze reactions upstream of lycopene biosynthesis in the carotenoid biosynthesis pathway. Lycopene levels are also affected by lycopene β-cyclases, which catalyze the conversion of lycopene into β-carotene, thus reducing lycopene accumulation. *Phytoene synthase 1* (*Psy1*) encodes an enzyme involved in the carotenoid biosynthesis pathway upstream of lycopene biosynthesis and Psy1 levels are regarded as rate limiting for lycopene production during ripening [37]. *Psy1* was upregulated specifically during ripening in wild-type fruits but was strongly repressed in *rin/rin* fruits (Fig 4). By contrast, *Psy1* was upregulated in *rinG2/rinG2* fruits compared with *rin/rin* fruits, supporting the differences in carotenoid accumulation between the *rin/rin* and *rinG2/rinG2* mutants. *Psy1* was expressed at substantial but lower levels in the two heterozygous mutants vs. the wild type and at slightly higher levels in *rinG2/+* fruits than in *rin/+* fruits, which was in accordance with the carotenoid levels in each line.

Of the several genes encoding lycopene β-cyclase, *Lycopene beta-cyclase 1* (*LcyB* or *CrtL1*) is indispensable for chloroplast activity during the vegetative growth phase but its expression is considerably reduced during ripening in wild-type fruits (Fig 4) [23]. Low levels of lycopene and high levels of β-carotene accumulate in *rinG2/rinG2* fruits due to high levels of *CrtL1* expression and reduced *Psy1* expression during ripening [23]. *CrtL1* was expressed at a lower level in *rin/+* fruits than in wild-type or *rinG2/+* fruits during ripening (Fig 4), which is consistent with the β-carotene levels in these lines.

### Ethylene biosynthesis in *rinG2*/+ fruits

We harvested mature green fruits from each line and monitored ethylene production from the fruits daily (Fig 3C). Wild-type fruits showed an initial increase in ethylene production when the fruit exhibited the first sign of red pigmentation or just before coloring; this ethylene production increased rapidly and reached a peak after 5 days. By contrast, we detected little or no increase in ethylene production in *rin* fruits throughout the assay. Ethylene production in fruits with the *rinG2* mutation began at the time of fruit pigmentation. The mutant fruits produced very low but significant levels of ethylene, and the fruits turned orange. Ethylene production in *rin/+* fruits rapidly increased, as observed in the wild type, but peak levels were ~72% that of the wild type. The *rinG2/+* fruits also showed a clear increase in ethylene production associated with red fruit coloring. However, a milder increase in production was detected compared to *rin/+* fruits, and the peak level was ~44% that of wild-type fruits.

1-Aminocyclopropane-1-carboxylic acid synthase (ACS) is a rate-limiting enzyme for ethylene production. Among the homologous genes encoding ACS, *ACS2* and *ACS4* are up-regulated at the onset of ripening and are thus responsible for the ethylene burst during ripening (Fig 4) [38, 39]. *ACS2* was expressed at substantial levels in the fruits of both heterozygous lines and even in *rinG2/rinG2* fruits, suggesting that *ACS2* expression is not sufficient for ethylene production. *ACS4* expression appeared to be more closely related to ethylene production in the lines.

## Discussion

The heterozygous effects of ripening mutations have been used to extend the shelf life of tomato fruit [6, 13]. Of the major ripening mutations, *rin* and *nor* show effects as heterozygotes, indicating that they partially inhibit the ripening process by substantially reducing ethylene production and softening rates while only slightly reducing lycopene accumulation [13]. Another ripening mutation is the dominant allele *Cnr* [40]; heterozygous *Cnr* inhibits ripening in a more severe manner than *rin* or *nor*, strongly reducing lycopene and β-carotene accumulation, as well as ethylene production [13]. Similar to *rin* and *nor*, the *rinG2* mutation had a semi-dominant effect on ripening, although *rinG2/+* fruits exhibited milder phenotypes than the classic ripening mutants. In *rinG2/+* fruits, fruit softening was delayed, but to a slightly lesser extent than in *rin/+* fruits, while carotenoids, especially β-carotene, accumulated to higher levels compared to *rin/+* fruits. These results suggest that F_1_ breeding using the *rinG2* mutation could be a useful option for improving shelf life when focusing on carotenoid content.

The targeting of genes involved in cell wall modification has been extensively investigated as a method to delay fruit softening in tomato [4, 41]. Silencing of genes encoding pectin methylesterase (PME), β-galactosidase (β-Gal), and expansin did not have a major impact on softening [42–45]. By contrast, silencing of *PL*, encoding an enzyme with pectin depolymerizing activity, substantially reduced fruit softening [35, 36], highlighting the important effect of pectin depolymerization on fruit softening. Another pectin depolymerizing enzyme, PG, is not a major contributor to tomato softening based on the results of gene silencing [34, 46, 47] and forced *PG* expression in *rin* fruits [48], suggesting that PG alone does not have sufficient activity for fruit softening. In the current study, however, *PG2a* expression was higher the fruits with the highest softening rates, including fruits of wild type, the *rin* and *rinG2* mutants, and the two heterozygous mutant lines, whereas *PL* expression in the *rinG2* mutant and the heterozygous lines was not likely correlated with fruit softening. These results suggest that PG activity may be rate limiting for softening only in genetic backgrounds including *rin* or *rinG2*, indicating that additional factors might be involved in PG activity-dependent softening. Genes showing inhibited expression in the *rin* or *rinG2* background, similar to the expression pattern of *Cel2* (Fig 4), might represent additional factors that promote softening in conjunction with PG activity. Studies of the ‘Delayed Fruit Deterioration’ tomato cultivar, which shows prolonged fruit firmness, suggested that (in addition to cell wall disassembly) water permeability through the fruit cuticle layer likely affects fruit softening [1, 8]. Analyses of the effects of *rin* and *rinG2* on permeability may find other key mechanisms for the reduced softening rate in the heterozygous mutants.

The *rin* and *rinG2* alleles produced different effects on ripening-associated ethylene production (Fig 3C). The activity of ACS isozymes is rate limiting for ethylene production, but ethylene production was not always correlated with *ACS2* and *ACS4* transcript levels in the *rinG2*-homozygous/heterozygous lines (Figs 3C and 4). The enzyme activity of ACS2 is regulated by a posttranslational modification, whereas ACS4 activity is not [49, 50]. In addition to ACS4 activity, ethylene biosynthesis in the two heterozygous lines might be regulated by the ACS2-activating system, which is unlikely to function in *rinG2/rinG2* fruits. The *rinG2/+* fruits produced lower levels of ethylene than *rin/+* fruits, although ripening physiology, including carotenoid accumulation and fruit softening, appeared to be more pronounced in *rinG2/+* fruits. The amount of ethylene required for ripening-associated gene expression might be significantly lower than that produced in wild-type fruits, as suggested by analysis of *PG* expression in *ACS*-antisense transgenic tomato plants [51, 52]. Ethylene production in *rinG2/+* fruits might be above the threshold required for ripening induction, but other factors, such as the transcription factor activities of the proteins encoded by the *RIN* alleles, might have stronger effects on ripening, leading to the differences between the two heterozygous lines.

Instead of F_1_ breeding, as performed in the current study, we previously tried to improve lycopene accumulation in *rinG2* homozygous fruits by knockout the *CrtL1* gene, which encodes an enzyme involved in metabolizing lycopene into β-carotene. The *CrtL1* experiment, however, produced albino plants because *CrtL1* is required for photosynthesis in green tissues, and thus the trial failed [22]. However, the *old gold* (*og*) and *old gold crimson* (*og*^*c*^) mutations were successfully used to increase lycopene accumulation in tomato. These mutants harbor a functional defect in the gene encoding a lycopene β-cyclase in the *Beta* (*B*) locus, which is a homolog of *CrtL1* [53]. Unlike the *CrtL1* knockout, the *og* and *og*^*c*^ mutations have no effect on vegetative growth. The use of the *og* or *og*^*c*^ mutation might be effective for improving lycopene accumulation in *rinG2* tomatoes.

In the current study, *rinG2* heterozygous fruits showed intermediate phenotypes between wild type and the *rinG2* mutant, indicating that *rinG2* has a semi-dominant effect on the wild-type allele. The effects of *rinG2* on counteracting the wild-type allele appear to be milder than the effect of the *rin* mutation. We previously determined that the phenotypes of F_1_ hybrid fruits between wild type and a *RIN-*knockout mutant were similar to the wild type in terms of fruit softening, carotenoid accumulation, and ethylene production, indicating that the knockout allele is recessive and, more importantly, that the dose of the wild-type allele in heterozygous fruits has little effect on ripening [22]. This finding suggests that the partial inhibition of ripening found in *rin-* and *rinG2-*heterozygous fruits was likely not due to the dosage effect of the wild-type allele but was instead due to the effects of the gene products of the mutant alleles, which accumulated in heterozygous fruits (Fig 1D) and have some transcription factor activity [22, 23]. The *rin* allele encodes a fusion protein between RIN and MC containing the DNA binding domain from RIN and the transcriptional repressor domain from MC but lacking the transcriptional-activating domain of RIN [9, 22]. The repressor activity of the rin protein is strong enough to inhibit the expression of most ripening-associated genes in the homozygous mutant (Fig 4) [15]. By contrast, the protein encoded by the *rinG2* allele has neither transcriptional activation nor repression activity, but it still binds to target genome regions by forming complexes with ripening regulating MADS-box proteins [TOMATO AGAMOUS-LIKE1 (TAGL1) and FUL homologs] in a similar manner to the wild-type RIN protein [23]. In *rinG2* homozygous fruits, the mutant allele had inconsistent effects on the transcription of ripening genes: some genes (such as *PG2a* and *Cel2*) were repressed in this mutant, but some genes (such as *FUL1*, *ACS2*, and *PL*) were expressed at substantial levels (Fig 4). The different effects of the two mutant alleles, however, appeared to become inconspicuous when these mutations were heterozygous with the wild-type allele, as these fruits showed partial softening and red pigmentation due to the partial reduction of ripening gene expression. Because the *rinG2* mutant protein forms complexes with other MADS-box proteins in a similar manner to wild-type RIN, in heterozygous condition, the rinG2 protein and the wild-type protein might compete for partner MADS-box proteins, suggesting that a certain amount of wild-type RIN may fail to be incorporated into transcription factor complexes. By contrast, rin mutant protein forms a homodimer but likely does not form complexes with the partners of RIN for DNA binding [22, 23]. Therefore, in *rin* heterozygous fruits, wild-type RIN forms complexes with its MADS-box partners without competition from the rin protein (unlike the situation in *rinG2* heterozygous cells), suggesting that more wild-type RIN may be incorporated into the transcription factor complex in an active form in *rin*/+ fruits compared with *rinG2*/+ fruits. In addition to competing for complex formation, the rin and rinG2 proteins may also compete with RIN for DNA binding sites in heterozygous fruits. The rin protein homodimer competes with wild-type RIN for binding to the *cis*-regulatory regions of the target ripening genes. The transcription of these genes is intensely inhibited by the binding of the rin protein. By contrast, in *rinG2* heterozygous fruits, the rinG2 protein might only counteract the transcriptional activity of the wild-type RIN protein to a minor extent, although the proteins compete for binding to target genome regions. Taken together, our findings suggest that despite the similar phenotypes of *rin-* and *rinG2-*heterozygous fruits, the rin and rinG2 mutant proteins might have different competitive effects on wild-type RIN during the formation of MADS-box protein complexes and the binding to target genome regions. Additional studies to obtain a more complete understanding of the molecular functions of these proteins will expand the potential for improving tomato fruit physiology and facilitate tomato breeding.

## Supporting information

S1 Fig Fruit appearances after long storage. (DOCX)

S1 Table Primers used in this study. (DOCX)

S1 Raw image The raw image of Fig 1D showing an immunoblotting assay. (PDF)
