# Supplementary material for: Semi-dominant effects of a novel ripening inhibitor (rin) locus allele on tomato fruit ripening
Source: PLoS One. 2021 Apr 22;16(4):e0249575. doi: 10.1371/journal.pone.0249575 (PMC8061929; doi:10.1371/journal.pone.0249575)
Supplement: S1 Fig — (DOCX) [file pone.0249575.s001.docx]

**
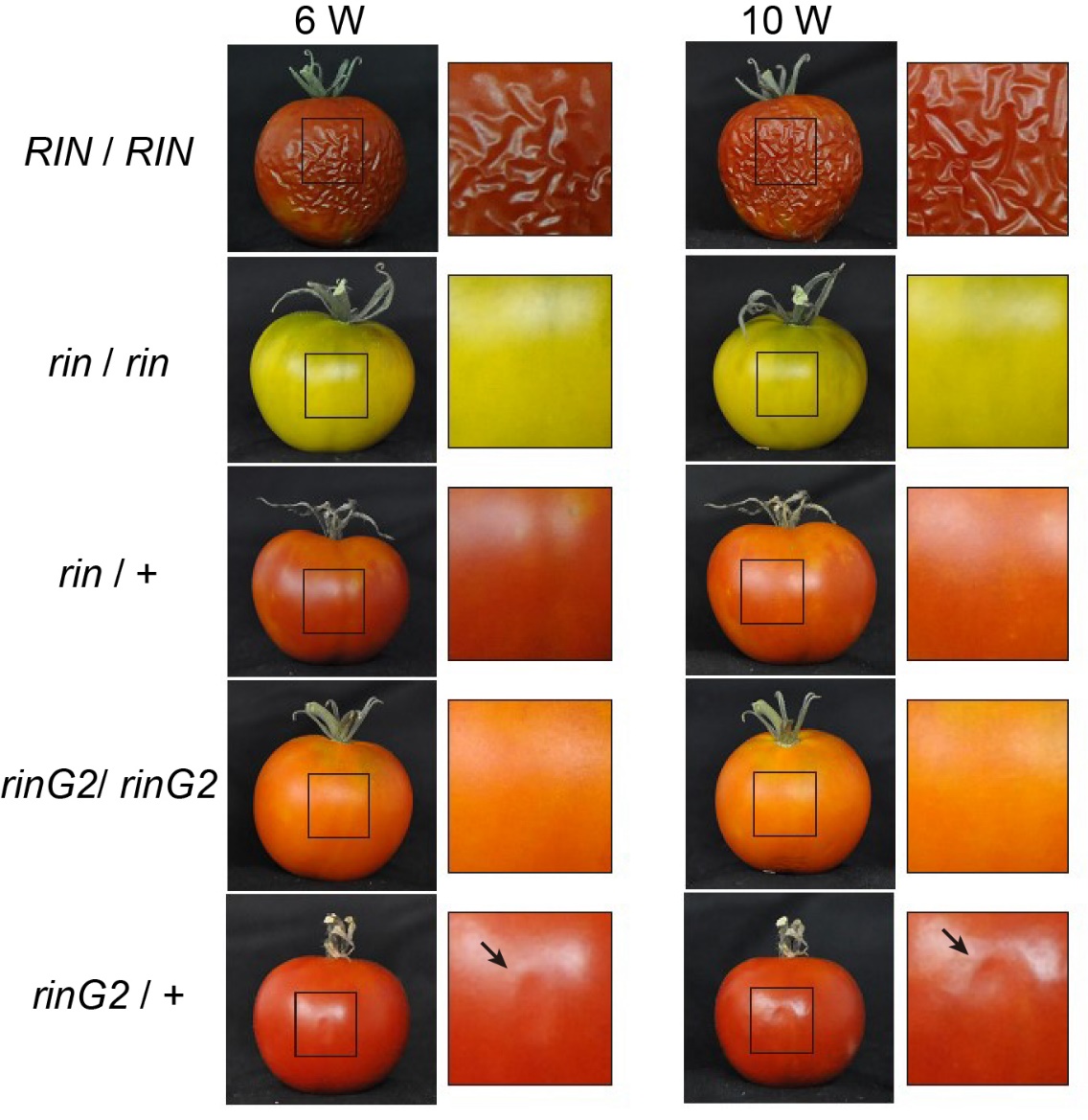
S1 Fig. Fruit appearances after long storage.** After 6 weeks storage, the surface of *rinG2/*+ fruits began to wrinkle. The process was, however, delayed in comparison with that found in the wild type fruits and showed less severe appearance. Enlarged images of Fig. 2 are shown.
