# Supplementary material for: Semi-dominant effects of a novel ripening inhibitor (rin) locus allele on tomato fruit ripening
Source: PLoS One. 2021 Apr 22;16(4):e0249575. doi: 10.1371/journal.pone.0249575 (PMC8061929; doi:10.1371/journal.pone.0249575)
Supplement: S1 Table — (DOCX) [file pone.0249575.s002.docx]

**S1 Table. Primers used for this study.**

| Gene | Gene ID |  | Primer sequence (5’ to 3’) |
| --- | --- | --- | --- |
| *RIN* alleles | Solyc05g012020 | F | GCTAGGTGAGGATTTGGGACAA |
|  |  | R | AATTTGCCTCAATGATGAATCCA |
| *FUL1* | Solyc06g069430 | F | CAACAACTGGACTCTCCTCACCTT |
|  |  | R | TCCTTCCACTTCCCCATTATCTATT |
| *ACS4* | Solyc05g050010 | F | AAATCTCCACTTTCACTAACGAACAT |
|  |  | R | TTGATAATTTCAGCGATGCTAACG |
| *ACS2* | Solyc01g095080 | F | GCACCACTTTGGACAAAGACAC |
|  |  | R | CCTGTTCATCGAGGATTTCAGC |
| *PSY1* | Solyc03g031860 | F | CCCTGTATGGGCATCTTTGG |
|  |  | R | TGATTTGCTCACATATGCTCTCTTT |
| *CrtL1* | Solyc04g04190 | F | GTGGATCTTGCTGTGGTTGGT |
|  |  | R | AAGCAGGACTCTCTGTTTGTTCAAT |
| *PG2a* | Solyc10g080210 | F | AGAATTTGCTCATGATTTTCAAGC |
|  |  | R | TGTTTTTCCATCACCCTTAGCTC |
| *PL* | Solyc03g111690 | F | GCGATCAGGAGTTAGAACTGG |
|  |  | R | AATCCCCTTTTGCTTTGGTT |
| *Cel2* | Solyc09g010210 | F | ACCTGGGGTTTGTAACATGCAG |
|  |  | R | GACGTTTGGCAATGTGTTTGAG |
| *CAC* | Solyc08g006960 | F | CCTCCGTTGTGATGTAACTGG |
|  |  | R | ATTGGTGGAAAGTAACATCATCG |
