# Supplementary material for: Semi-dominant effects of a novel ripening inhibitor (rin) locus allele on tomato fruit ripening
Source: PLoS One. 2021 Apr 22;16(4):e0249575. doi: 10.1371/journal.pone.0249575 (PMC8061929; doi:10.1371/journal.pone.0249575)
Supplement: S1 Raw image — (PDF) [file pone.0249575.s003.pdf]

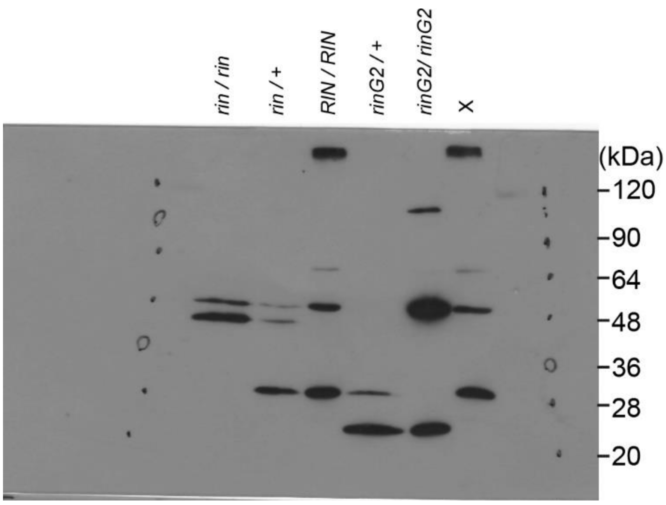

S1 raw image. This is a raw image of Fig. 1D showing an immunoblotting assay. Proteins from nuclei of fruits harvested at 4 days after the breaker stage were separated and subjected to an immunoblotting assay with RIN-antibodies with RIN-antibodies. The lane indicated as “X” was not included in Fig. 1D but was an duplicate assay for the fruits with the *RIN/RIN* genotype. Immunodetection reactions were performed on membranes with antiserum against RIN as primary antibodies and horseradish peroxidase (HRP)-linked anti-rabbit IgG (GE Healthcare) as secondary antibody. Fluorescence signals generated with the Chemi-Lumi One L (Nacalai Tesque) were detected by exposure to X-ray film, and the film image was scanned.
